# Lateral Supracondylar Spur Process of the Humerus

**DOI:** 10.7759/cureus.13514

**Published:** 2021-02-23

**Authors:** Somya Bhatnagar, Joe Iwanaga, Aaron S Dumont, R. Shane Tubbs

**Affiliations:** 1 Neurosurgery, Tulane University School of Medicine, New Orleans, USA; 2 Neurosurgery and Structural & Cellular Biology, Tulane University School of Medicine, New Orleans, USA

**Keywords:** supracondylar process, struthers, humerus, variant, anatomy

## Abstract

The supracondylar process is a rare but commonly reported anatomical variant of the humerus. Though it is usually asymptomatic, it can lead to serious symptoms. In this report, a lateral supracondylar process of the humerus was found. This is much rarer than a medial supracondylar process, and to our knowledge, it has not been reported previously. Surgeons and radiologists must account for supracondylar process variations to diagnose neurovascular pathology in the forearm accurately and quickly to optimize surgical outcomes. Here, we describe the origin and clinical importance of the supracondylar process variant.

## Introduction

The supracondylar process, or spur, is a rare bony projection on the distal, medial portion of the humerus. The ligament of Struthers connects the process inferiorly to the medial epicondyle of the humerus. It usually occurs unilaterally 5-7 cm superior to the medial epicondyle. The supracondylar process is clinically relevant because it forms a passage with the humerus and the ligament of Struthers. The median nerve and brachial artery, which pass through this channel, can become compressed leading to symptoms of neurovascular impingement. These symptoms are exacerbated when the elbow is hyperextended or pronated [1].

Supracondylar processes are normally present in climbing mammals such as cats and lower primates. They form the roof of a tunnel that encompasses the artery and nerve in the forearm. The ligament of Struthers, inserting on the tip of the process, is part of a tendon of a vestigial muscle called the latissimo-condyloideus, usually present in climbing mammals. In humans, it is a fibrous band between the tendons of the latissimus dorsi and coracobrachialis [2].

We discovered a lateral supracondylar process of the humerus, a much rarer anatomical variant than the medial supracondylar process. This case illustrates the distinctive morphologies of the humerus. The location of the more commonly reported “medial” supracondylar process near the median nerve and brachial artery can produce neurovascular symptoms. Because the process is usually asymptomatic, it is only discovered incidentally during radiographic imaging. Knowledge of this variant can prevent misdiagnosis in radiology and improve symptoms through surgical excision.

## Case presentation

**Figure 1:**
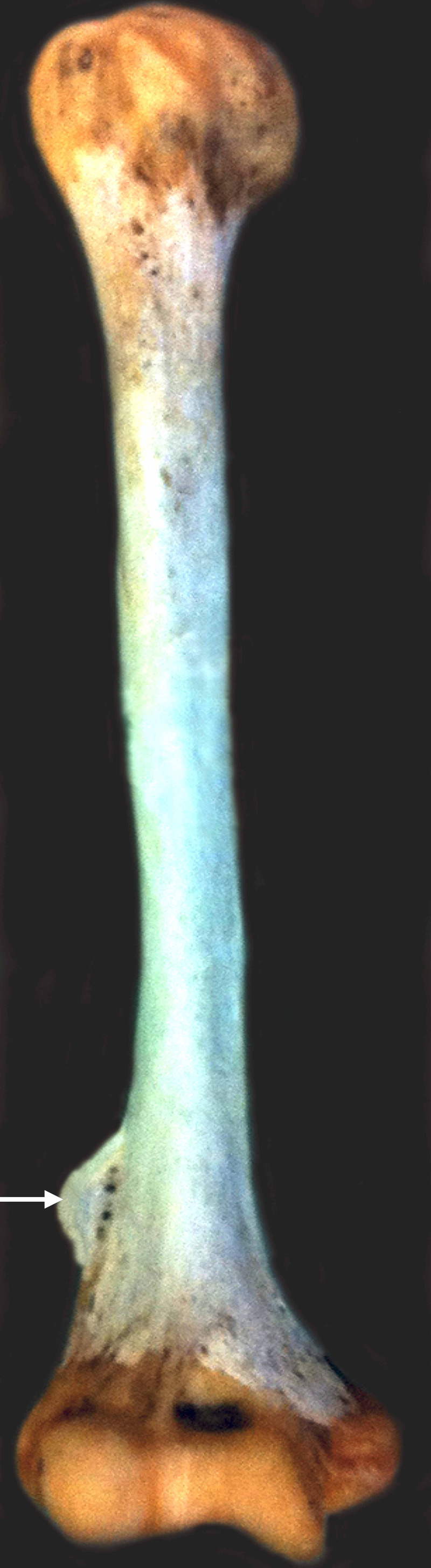
Right humerus with the lateral supracondylar process (arrow).

**Figure 2:**
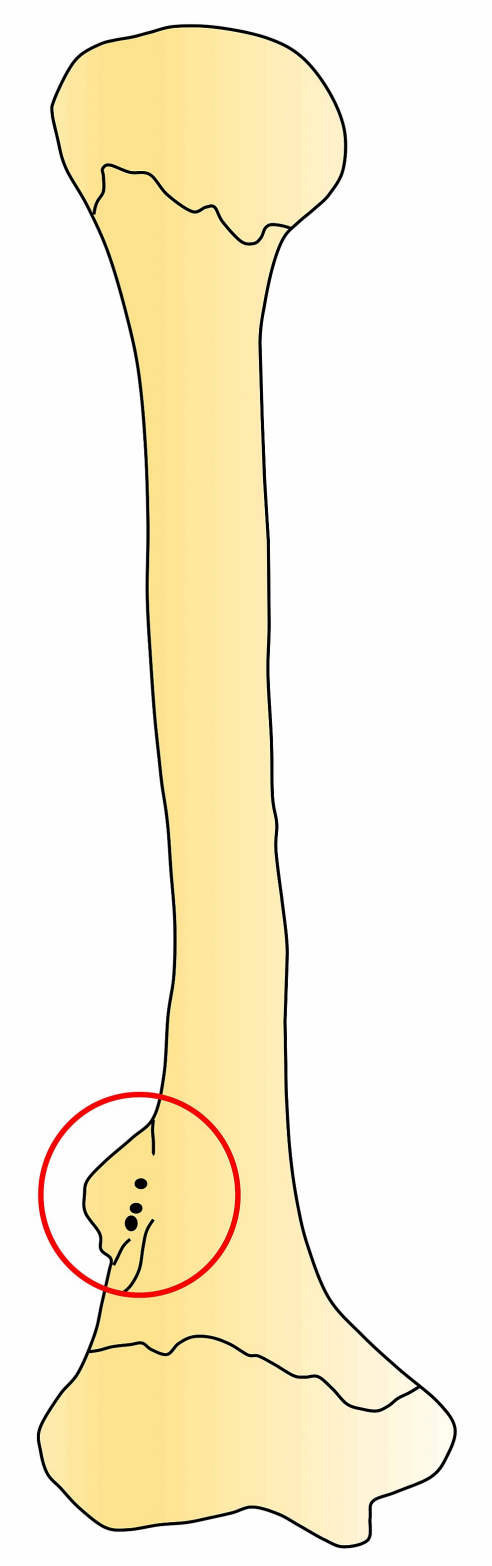
Schematic drawing of the present case. The circle shows the lateral supracondylar process.

During routine evaluation of an isolated adult humerus, an unusual bony variant was noted. A lateral supracondylar process of the right humerus was found arising proximal to the lateral epicondyle by about 2 cm (Figures 1, 2).

 

The process was roughly 2.5 cm long and 1 cm wide and fin-shaped. The thickness of the process was uniform and approximately 1 mm. At the base of the process where it attached onto the humerus, there were several small bony foramina. The remaining bony architecture of the humerus was found to be within normal limits. There were no signs of pathology such as bony hyperplasia. As an isolated bone, the remaining skeleton that this specimen originated from was not available for study.

## Discussion

The supracondylar process, arising from the medial aspect of the distal humerus, occurs in 0.3%-2.7% of the human population and is usually asymptomatic. Any symptoms that occur depend on the degree of compression of the median nerve, ulnar nerve, and brachial artery, resulting in supracondylar process syndrome. Bilateral involvement is exceedingly rare [3]. The most common cases involve compression of the median nerve as it passes under the ligament of Struthers. In the relatively few cases that involve the ulnar nerve, the nerve stretches posteriorly around the process but does not pass under the ligament of Struthers. The brachial artery or its branches are typically compressed under the ligament of Struthers resulting in ischemic symptoms such as forearm claudication and cyanosis. A process proximal to the medial epicondyle can also lead to carpal tunnel syndrome-like symptoms, ulnar nerve symptoms, loss of sensation, and disappearance of the radial or ulnar artery pulse on extension and supination of the forearm [4]. Ivins describes a case of a median nerve and brachial artery compressed by the ligament of Struthers. The supracondylar process also impinged on the ulnar nerve. Resection of the supracondylar process and ligament of Struthers relieved the symptoms quickly [5]. Pain and hand numbness were the two symptoms reported by Shon et al. [6] in two patients presenting with a supracondylar process.

Our case appears to be unique in that the process arose laterally. Based on its location, we would speculate that it might represent an ossified lateral intermuscular membrane, although no soft tissues were attached to this dry bony specimen. Over time, stress to this region of the humerus by the nearby muscles such as the brachialis or brachioradialis directly or indirectly via the lateral intermuscular septum might result in the ossification seen in the present case. In this position, the radial nerve or nearby arteries such as the radial collateral artery could potentially be compressed by such a bony irregularity. The genetics of such a rare bony variation of the humerus are not clear, but perhaps, with additional cases, this can be clarified.

Talha et al. describes a case of a supracondylar process compressing the brachial artery owing to an unusual attachment of the pronator teres muscle above its typical point of insertion [7]. The patient experienced distal paresthesia of the fingers with an elbow in hyperextension. Symptoms were reduced upon flexion. It was first believed that the patient had thoracic outlet syndrome. Despite resection of the first rib, the symptoms persisted. It was later found that hyperextension of the elbow eliminated the patient’s radial pulse and the brachial artery was shifted medially, stopping blood flow. During surgery, a muscle sheet was found along the medial portion of the humerus, attaching 6 cm above the medial epicondyle. Upon dissection of the muscle fibers, which inserted on a small bony projection on the humerus, the brachial artery was seen underneath. Although the symptoms suggested thoracic outlet syndrome, it was discovered that the brachial artery was entrapped by the supracondylar process.

Supracondylar fractures are among the most common types of fractures in children. Stress fractures of the supracondylar process are difficult to treat owing to its association with nerves and vessels. Therefore, knowledge of this variant can reduce complications during surgery [8]. The supracondylar process is also associated with Cornelia de Lange syndrome, caused by an autosomal recessive trait. The syndrome results in mental retardation, dwarfism, and skeletal changes. Curtis et al. reported a unilateral supracondylar process in three of the six cases of Cornelia de Lange; this process has not been associated with any other syndrome [9].

The supracondylar process can mimic exostosis and osteochondromas. However, it is located distally toward the elbow, with a continuous cortex, while an osteochondroma points away from the joint. Knowledge of this difference will reduce misdiagnosis by radiologists [8,10]. When the supracondylar process is ossified, it can be seen on radiographs, though not always [7]. As the lateral view does not always reveal the process, the anteroposterior or oblique view can be better for demonstrating it, especially due to ossification densities. Clinicians should consider the presence of a supracondylar process in the differential diagnosis of patients with neurovascular symptoms.

The authors sincerely thank those who donated their bodies to science so that anatomical research could be performed. Results from such research can potentially increase mankind’s overall knowledge that can improve patient care. Therefore, these donors and their families deserve our highest gratitude [11].

## Conclusions

Anatomical variants of the supracondylar spur must be kept in mind when diagnosing patients with symptoms of neurovascular compression in the forearm. Clinicians and surgeons must account for supracondylar spur variants to accurately diagnose impingement pathology, interpret radiological images, and optimize surgical outcomes.
